# Prediction of Successful Memory Encoding Based on Lateral Temporal Cortical Gamma Power

**DOI:** 10.3389/fnins.2021.517316

**Published:** 2021-05-25

**Authors:** Soyeon Jun, June Sic Kim, Chun Kee Chung

**Affiliations:** ^1^Department of Brain and Cognitive Sciences, Seoul National University, Seoul, South Korea; ^2^Research Institute of Basic Sciences, Seoul National University, Seoul, South Korea; ^3^Department of Neurosurgery, Seoul National University Hospital, Seoul, South Korea

**Keywords:** memory prediction, successful memory encoding, electrocorticography, high-frequency activity, memory formation, gamma frequency

## Abstract

Prediction of successful memory encoding is important for learning. High-frequency activity (HFA), such as gamma frequency activity (30–150 Hz) of cortical oscillations, is induced during memory tasks and is thought to reflect underlying neuronal processes. Previous studies have demonstrated that medio-temporal electrophysiological characteristics are related to memory formation, but the effects of neocortical neural activity remain underexplored. The main aim of the present study was to evaluate the ability of gamma activity in human electrocorticography (ECoG) signals to differentiate memory processes into remembered and forgotten memories. A support vector machine (SVM) was employed, and ECoG recordings were collected from six subjects during verbal memory recognition task performance. Two-class classification using an SVM was performed to predict subsequently remembered vs. forgotten trials based on individually selected frequencies (low gamma, 30–60 Hz; high gamma, 60–150 Hz) at time points during pre- and during stimulus intervals. The SVM classifier distinguished memory performance between remembered and forgotten trials with a mean maximum accuracy of 87.5% using temporal cortical gamma activity during the 0- to 1-s interval. Our results support the functional relevance of ECoG for memory formation and suggest that lateral temporal cortical HFA may be utilized for memory prediction.

## Introduction

Memory formation is an important cognitive process that enables the identification of traces of individual episodic memories and learning from experiences to guide behavior (Chadwick et al., 2010). Understanding the neural correlates of memory formation is essential to identify the brain mechanisms underpinning memory processes, which can be further applied to predict subsequent memories or even improve memory (Ezzyat et al., 2017). The decoding of neural activity during memory processing has garnered substantial interest in the cognitive neuroscience community. Neural activity relevant to memory formation measured with electrocorticography (ECoG) provides a valuable window into the neural correlates of underlying cognitive processes (Fell et al., 2011). The field potential of ECoG activity interacts with neural membrane potentials and, thus, modulates the degree of neuronal excitability and influences their discharge times (Anastassiou et al., 2010; Hohne et al., 2016). As such, these studies have provided evidence for the role of the amplitude of cortical oscillatory activities in neural processing.

There has been growing interest in human brain oscillations and their possible role in memory processes. Low-frequency activity (i.e., theta rhythm, 4–8 Hz) and high-frequency activity (HFA) (i.e., gamma rhythm, >30 Hz) have received attention in the context of understanding human memory function (Sederberg et al., 2003, 2007; Kahana, 2006). In particular, HFA is a brain response with ECoG signals for episodic memory formation, which provides spatiotemporal properties of memory encoding with millisecond temporal resolution. The neural substrates that produce such fast activity is a topic of ongoing research. HFA has been linked to asynchronous signals related to increased multi-unit activity (Manning et al., 2009; Milstein et al., 2009; Ray and Maunsell, 2011). An increasing number of studies have leveraged HFA as a marker of underlying neural activation (Miller et al., 2008; Shenoy et al., 2008; Lachaux et al., 2012), and HFA is, thus, considered to reflect regional activation during memory encoding (Burke et al., 2014). HFA has been reported to be a potential biomarker for mapping, targeting, and modulating neuronal assemblies at a high temporal resolution during memory formation (Lachaux et al., 2012; Burke et al., 2015; Johnson and Knight, 2015). In particular, these oscillations spanning a 30- to 150-Hz range were proposed to set an ideal frame for neuronal interactions underlying memory formation (Jensen et al., 2007; Duzel et al., 2010). Thus, studies investigated to detect discrete events induced during memory formation of word encoding in different gamma band activities—low gamma (30–60 Hz) and high-gamma (>60 Hz) (Colgin et al., 2009; Buzsaki and Silva, 2012). Separating different types of gamma activities (30–150 Hz) is a crucial electrophysiological biomarker of memory formation and applications (Kucewicz et al., 2017).

Extant evidence suggests that prevalent HFA from structures outside the medial temporal lobe (MTL) is critical for memory formation (Buzsaki, 1996; Eichenbaum, 2000; Poldrack et al., 2001; Ritchey et al., 2015; Moscovitch et al., 2016). Neuroimaging studies have provided evidence for the neural correlates of episodic encoding within the hippocampus and functional networks spanning prefrontal, medial temporal, lateral temporal, and parietal cortical regions (Kim et al., 2010). Similarly, successful memory processing relies on coherent oscillations of multiple temporal and neocortical regions at varying frequencies. For instance, increased coherence between brain regions, particularly the hippocampus and prefrontal cortex, is associated with better memory (Fell et al., 2008; Benchenane et al., 2010; Watrous et al., 2013). Especially, gamma oscillatory power increases with memory task in the hippocampus, and this gamma pattern (28–40 Hz and 90–100 Hz) was observed in other memory-related regions such as frontal and temporal cortical regions (van Vugt et al., 2010). The neural correlates of HFA of successful memory processing in neocortical regions may, therefore, provide insight into the roles of specific regions in memory performance, and characterizing these features may facilitate the evaluation of memory performance. However, the effect of HFA in human ECoG signals to differentiate memory prediction has been little explored.

The core aim in this study is to provide novel evidence on how HFA in the temporal cortex is associated with success of memory formation in human ECoG signals and to differentiate memory process into remembered and forgotten memories with HFA. We evaluated temporal cortical HFA, which was accompanied by successful memory formation relative to unsuccessful encoding. We hypothesized that the difference in HFA would enable differentiation of successful encoding trials from unsuccessful ones. In the first step, we identified time windows (i.e., pre-stimulus vs. during-stimulus) referenced by the human single-unit activity and HFA with statistically significant power clustering across subjects. We delegated the HFA to low gamma (30–60 Hz) and high gamma (60–150 Hz) based on previous literature, revealing a sequential memory effect (SME) during the encoding phase (Sederberg et al., 2007; van Vugt et al., 2010). We then determined the brain regions and frequencies for which the amplitude differences differed between the remembered and forgotten conditions in order to analyze encoding-related activities for subsequently remembered and forgotten words. Finally, a support vector machine (SVM) was trained using the power in the selected time windows and frequencies.

## Materials and Methods

### Subjects

The present study included six subjects (four women; mean age: 34.2 ± 11.6 years) with drug-resistant epilepsy who had been implanted with intracranial electrodes to determine the area of the seizure onset zone. The local institutional review board (IRB) approved the study protocol (H-1407-115-596). All subjects provided written informed consent to participate in the present study. Subject characteristics are presented in Table 1. Most of the subjects underwent neuropsychological assessments including IQ and MQ to confirm that the subjects were within a normal cognitive category.

**TABLE 1:** Subject demographics, clinical characteristics, and electrode locations.

**Subject**	**Demographics**		**Clinical characteristics**				
	**Age**	**IQ/MQ**	**Seizure onset**	**Pathology**	**Resection**	**Seizure type**	**Electrode Type**
Sub1	50–55	77/94	ATG, TP	PHG reactive gliosis	PHG	TLE	Subdural
Sub2	30–35	N/A	TP, STG	Temporal lobe Focal cortical dysplasia	L. ITG	Bilateral TLE	Subdural
Sub3	20–25	89/92	Amygdala	FCD Heteropia	PHG, Amygdala	TLE	Subdural
Sub4	40–45	85/85	STG	HP neuronal loss	ATL, AH	TLE	Subdural
Sub5	25–30	N/A	PHG	DG dispersion, HP neuronal loss	HP	TLE	Subdural
Sub6	25–30	N/A	ATG, TP	PHG reactive gliosis	PHG	TLE	Depth

*IQ, intelligence quotient; MQ, memory quotient; R, right; L, left; HP, hippocampus; mHP, middle hippocampus; LWM, limbic white matter; PHG, parahippocampal gyrus; DG, dentate gyrus; aTG, anterior temporal gyrus; STG, superior temporal gyrus; ITG, inferior temporal gyrus; TP, temporal pole; TLE, temporal lobe epilepsy; FCD, focal cortical dysplasia; N/A, not applicable.*

*Subject demographic data are presented together with clinical observations from clinically identified seizure onset zones, pathology in subjects who underwent corresponding surgery and showed neuropsychological results. A clinical psychologist employed the Wechsler Adult Intelligence Scale—Korean version (K-WAIS-IV) and the MQ of the Rey–Kim Memory test to assess IQ. Most of the subjects underwent neuropsychological assessment including IQ and MQ, providing that the subjects were within a normal cognitive category.*

### Electrode Localization

**FIGURE 1:**
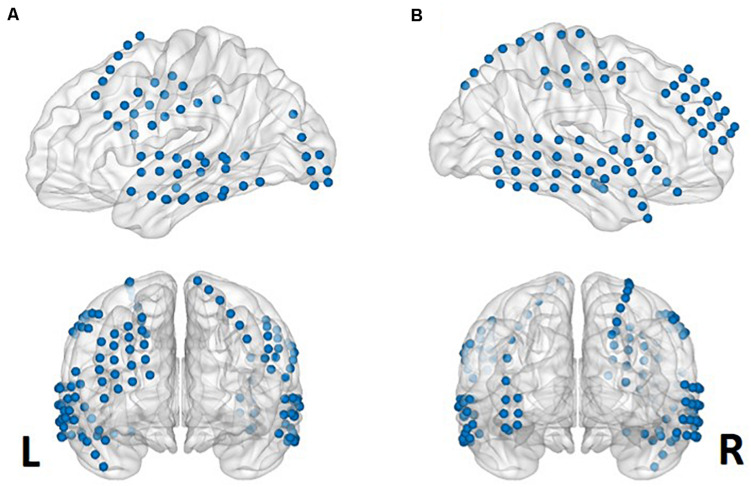
Aggregate electrode rendering. Grid electrodes from all six subjects rendered on normalized cortical surfaces. Lateral sagittal (upper) and coronal (lower) views from left **(A)** to right **(B)**.

The locations of the electrodes were determined by clinical diagnosis. The electrodes (AdTech Medical Instrument Corporation, Racine, WI, United States) were positioned for subdural electrocorticography (ECoG) on the cortical surface (diameter of 4 mm, placed 10-mm apart) with stainless steel contacts. Prior to electrode implantation, each subject underwent a preoperative magnetic resonance imaging (MRI) scan in a Magnetom Trio, Magnetim Verio 3-tesla (Siemens, München, Germany) or Signa 1.5-Tesla scanner (GE, Boston, MA, United States). Computed tomography (CT) scans were performed following electrode implantation using a Somatom sensation device (64 eco; Siemens München, Germany). For visualization, CT and MRI images were co-registered as previously described (Avants et al., 2008). The brain model and implanted electrodes were reconstructed from individual preoperative MRI and postoperative CT images using CURRY software version 7.0 (Compumedics Neuroscan, Charlotte, NC, United States) (Figure 1). A neuroradiologist and neurosurgeon performed electrode localization based on thin-section post-implantation CT scans and co-registered MR images. BrainNet Viewer (Xia et al., 2013) was used to visualize the electrodes.

### Verbal Memory Task

**FIGURE 2:**
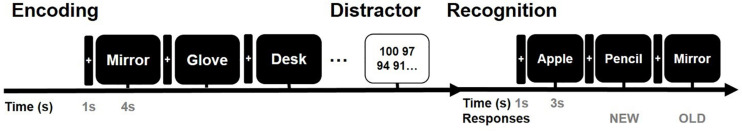
Verbal memory task paradigm. Example of the timeline of the word memory paradigm. The entire task consisted of three study periods: encoding, rest (distractor), and retrieval.

All stimuli were presented on a laptop computer with a Stim 2 Gentask (Neuroscan, Charlotte, NC, United States). We used a word memory task (Figure 2), which is known to recruit the medial temporal lobe during memory encoding (Axmacher et al., 2008; Hamani et al., 2008; Jun et al., 2020). All words consisted of concrete Korean nouns according to the Korean Category Norms: Survey on Exemplar Frequency Norm, Typicality, and Features (Rhee, 1991) and the second version of the Modern Korean Words database (Kim, 2005). Prior to the main experiment, a brief practice set of trials was conducted to ensure that the subjects understood the task. For

the task, subjects were instructed to memorize the presented words. The subjects were instructed to study 60 words across two sessions. Each session consisted of 30 words. In total, 60 concrete nouns were individually presented in a random manner. The presentation of each word commenced with a fixation cross appearing on the screen for 1 s during the pre-stimulus time period, followed by the word that was displayed for 4 s. To ensure deep encoding, subjects were instructed to report whether they judged the word on the screen as “pleasant” or “unpleasant” by pressing a keyboard button with their index finger (de Vanssay-Maigne et al., 2011). Following presentation of the final word of the encoding block, subjects were allowed a 10-min break and subsequently performed a 30 s distractor task consisting of a series of arithmetic problems for “A − B = ?” where A and B were randomly chosen integers ranging from 1 to 100. In the item task, a total of 90 words were used, including 30 new words and 60 old words. Subjects were instructed to respond whether the word had been presented before (“old”; button #1), new (“new”; button #2), or no idea (“no idea”; button #3). For the main experimental session, none of the words were presented twice, and subjects were not exposed to the same experimental task more than once.

### Data Acquisition and Analysis

ECoG and depth electrodes were recorded using a 64-channel digital video monitoring system (Telefactor Beehive Horizon with an AURA LTM 64- and 128-channel amplifier system; Natus Neurology, West Warwick, RI, United States) digitized at a sampling rate of 1,600 Hz and filtered from 0.1 to 150 Hz. These ECoG data were analyzed using MATLAB software (version 2015b, Mathworks, Natick, MA, United States). The depth electrode was implanted only in Subject 6, and it covered the temporal white matter. The depth electrode did not cover the region of interest in the present study, and we excluded the depth electrode from further analysis. We first performed manual artifact rejection of the signal for every electrode. Channels affected by artifacts were excluded from subsequent analyses. Individual stimulus response trials were marked and precluded if motion artifacts were present. Signals exhibiting motion artifacts and epileptic-form spikes were also marked and excluded from further analyses. The recorded data were re-referenced to the common average reference. To quantify specific changes in frequency bands during stimulation for the encoding period of the memory task, time-frequency analysis with Morlet wavelet transformation (wave number: 2.48) was applied to obtain a continuous-time complex value representation of the signal. The effective window length (95% confidence interval of the Gaussian kernel, seven cycles) was 80 ms at 50 Hz. Transformed data were squared to calculate the power value and normalized by the mean of the pre-stimulus baseline power (i.e., resting periods prior to the task) for each frequency. The resting periods prior to the memory task was 5-min duration, and it was equal to every subject. During the resting periods, the subjects were instructed to keep their eyes open, while fixating a white cross in the notebook. A fixation cross, on which subjects were instructed to focus their gaze, was presented to minimize eye movement. The electrophysiological data were divided into epochs that onset 1 s pre-stimulus and continued to 1 and 1.5 s of during stimulus from the onset of the word trials and sorted according to subsequent memory performance. The averaged power of each condition was compared across a frequency range of 30–150 Hz for correctly and incorrectly encoded memory items. Normalized data were averaged across all trials for correct and incorrect trials according to each condition. To test the significance between subsequently remembered and subsequently forgotten words at encoding, independent two-sample t-tests were performed.

### Feature Selection

**FIGURE 3:**
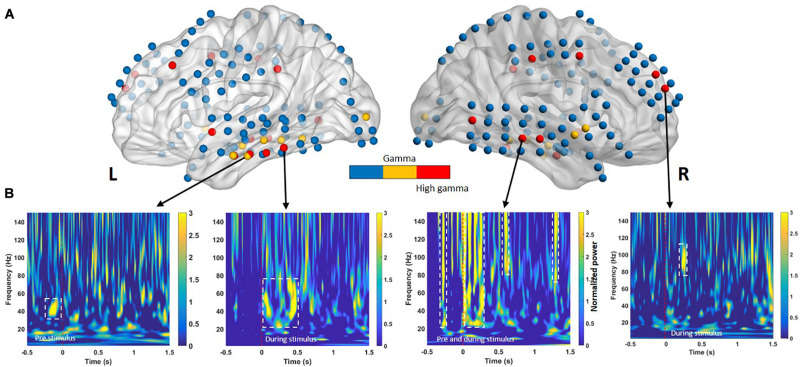
**(A)** Electrodes in cortical regions exhibiting across-subject differences between successful memory encoding (SME) for the −0.5- to 1.5-s time bins. The color intensity indicates the direction of the effect (yellow = low gamma, red = high gamma) with a significance threshold of *p* < 0.05. Blue denotes regions that did not exhibit a significant effect among subjects. **(B)** Time course of significant oscillatory activity in SMEs for lateral temporal cortices from four individuals (Subject 1 to Subject 4, left to right, respectively). Each panel shows the *t*-transformed significance value of the difference in power between remembered and forgotten memories. The left and right temporal cortices both exhibit heightened low-gamma power (30–60 Hz) and high-gamma oscillation (∼150 Hz) increases during pre- and during-stimulus intervals in SME. The dashed white line indicates *p* < 0.05 significance threshold.

Figure 3 presents the selected features for each phase and frequency band. Table 3 presents the t-statistic values and regions of the selected features. The most informative frequency values with the top 20% of t-statistics were selected as the features in each phase and frequency band.

### Classification Problem

The classification problem was set up. Trials that were presented in the encoding phase were labeled according to the results of recognition phase. Remembered and forgotten were labeled. There were two labels: remembered and forgotten. The remembered class consisted of trials where the subjects pressed the buttons “Old” (old words correctly recognized as old), and the forgotten class consisted of trials where the subjected pressed the buttons “New” (old words incorrectly recognized as new). Since all subjects only responded as “old” and “new,” we could not get “no idea” trials. Furthermore, new trials were not included to maximize the difference in encoding process. Sets of labeled trials were acquired from two different periods: pre- and during stimulus. These spectral classifier learned the power differences between the remembered and forgotten trials from the three separate time windows (i.e., −500 to stimulus onset, stimulus onset to 1 s, and stimulus onset to 1.5 s).

### Classification

For classification between remembered and forgotten trials from low- and high-gamma band signals, features from the single-trial low- and high-gamma power (dashed line in Figures 3A,B) of all electrodes located in Figures 3A,B were first extracted. The *p*-values were then calculated by comparing the remembered and forgotten items. To confirm whether the features based on the HFA difference in the single-trial conditions represented their respective successful memory encoding (SME), simple linear SVM analyses were performed. The selected feature sets were entered into a supervised linear classification procedure using an SVM algorithm to assess whether subsequently remembered trials could differentiate subsequently forgotten trials. A data-driven feature-filtering step was performed before SVM learning. The most informative power with statistical significance was within the high-frequency power (low gamma, 30–60 Hz; high gamma, 60–150 Hz) as identified using the subsequent memory effect (SME) procedure in the encoding phase (Sederberg et al., 2007; van Vugt et al., 2010). The most informative power was selected as a candidate feature for SVM learning to identify the optimal classifier modified from a previous study (Jin and Chung, 2017). SVM group classification analyses were performed using the Statistics Toolbox in Matlab software (version R2018b; MathWorks Inc., Natick, MA, United States). The nonlinear radial basis function kernel (sigma = 2) and constant soft margin (cost = 1) were applied for the SVM training, as recommended previously, showing high gamma time features with an SVM model that classified individual words from a pair of words (Martin et al., 2016). In the SVM training procedure, the decision boundary formulated using a candidate feature set was optimized to maximize group classification accuracy using 80% of trials randomly selected from the total trials (Dosenbach et al., 2010). All SVM procedures, testing, and iterative group classifier performance evaluation (with random permutation of subjects into training and testing sets for cross-validation) were repeated 10,000 times per candidate feature set. The most accurate group classifier with the highest overall mean accuracy across the 10,000 cross-validation procedures was selected as the optimal SVM group classifier.

### Statistical Analysis

Statistical tests were performed using the Statistical Package for Social Sciences v12.0 K (SPSS) and MATLAB (Mathworks). Our primary measurement of memory performance was the percentage of correctly recognized trials in each block. Paired non-parametric rank-sum tests were used to compare behavioral performance between conditions. For activity in the lateral temporal cortex, independent two-sample t-statistics (^∗∗^*p* < 0.01 or ^∗^*p* < 0.05) were used to compare the average power amplitudes of ECoG waveforms between correctly and incorrectly recognized trials. Prior to significance testing, normality was assessed using the Lilliefors test (*p* > 0.01, for all datasets). For multiple comparisons among gamma power levels, the Bonferroni correction procedure was employed. The level of statistical significance was set at *p* < 0.05.

## Results

On average, subjects successfully remembered 81.19 ±5.79% (standard error of the mean; SEM) of the words, with a mean response time of 1,277.45 ± 315.28 ms (1,076.26 ±181.82 ms for remembered trials and 1,478.64 ± 475.34 ms for forgotten trials, *p* > 0.05). Full-scale IQ (FSIQ) and memory quotation (MQ) were measured in six subjects before electrode implantation as part of the routine clinical preoperative evaluation. Subjects had an average preoperative FSIQ of 83 ± 8 (mean ± SEM) and MQ of 85.6 ± 8.45. No significant correlations were observed between preoperative FSIQ and accuracy during the task (*r* = −0.300, *p* = 0.624, N = 5) (*r* = −0.200, *p* = 0.747, N = 5) across all sessions for each subject, suggesting that task performance was associated with normal psychometric measurements.

### Temporal and Spectral Successful Memory Effects

Previous memory studies have compared signals during learning of visual items that are subsequently remembered to items that will be forgotten to assess differences in brain activity, yielding an outcome termed the SME. Positive and negative SMEs have been reported in different frequency bands (Hanslmayr et al., 2012). Interpretation of these effects suggests that the power increase for remembered items typically occurs in positive high-frequency SMEs (Sederberg et al., 2003; Burke et al., 2014, 2015). In our study, SMEs in the pre- and during-stimulus intervals were identified using the methods described in the *Classification* section. Oscillatory power in the pre- and during-stimulus intervals was examined separately for two nonoverlapping sub-bands (low gamma, 30–60 Hz; high gamma, 60–150 Hz). For a given sub-band, within-subject averages of the power difference between the remembered and forgotten trials were calculated for all electrode positions. An independent two-sample *t*-test was performed to identify differences in gamma power between the remembered and forgotten trials. Multiple comparisons confirmed that the during-stimulus period exhibited consistent positive spectral SME across subjects in the low- and high-gamma bands in the left and right temporal cortical electrodes, as shown in Figure 3B.

### Predictive Performance of Pre- and During-Stimulus Intervals

**FIGURE 4:**
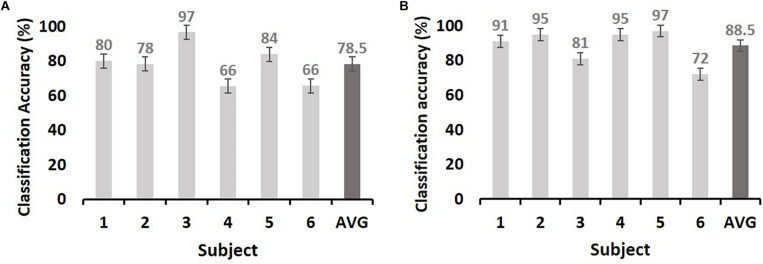
The maximum accuracy of successful memory classification by simple SVM using the pre-stimulus interval [**(A):** −0.5–0 s] and during-stimulus interval [**(B)**: 0–1 s]. The x-axis indicates subjects, and y-axis indicates the classification accuracy (%). The chance level is 50% (dashed line). The dark gray bars in both **(A)** and **(B)** indicate the average accuracy. Error bars indicate the standard error of the mean.

We next evaluated the type of ECoG signals that contributed to memory performance prediction. ECoG signals from the two different intervals were considered separately as input from all electrodes for the classification of statistical differences in gamma power from left to right hemispheres (Figure 3A; yellow and red dots, respectively). Performance during the pre-stimulus interval (−0.5 to 0 s) was compared with that for the first and second during-stimulus epochs (0–1 and 0–1.5 s, respectively) (Table 2), revealing the predictive accuracy and final included number of trials for each participant. The optimal SVM group classifier with the top 10 ranked features among the 20 significantly different frequency bands according to the averaged *t*-statistics distinguished correct versus incorrect trials. The overall predictive performance with pre-stimulus signals was 78.5% (averaged over six subjects) (Figure 4A) and that of the during-stimulus intervals was approximately 88.5% (Figure 4B) and 85.5% for the first and second epochs, respectively (averaged over six subjects). The accuracy of each subject was significantly greater than chance levels (50%) for the entire period. Compared with the average accuracy using the pre-stimulus interval of ECoG data, the average accuracy using the first epoch of the during-stimulus interval increased to 88.5%, which was similar to that for the second epoch of the during-stimulus interval.

**TABLE 2:** Prediction accuracy using two different periods of during stimulus.

**Subject**	**Pre-stimulus interval**	**During-stimulus interval**		**# trials (REM/FOR)**
	**−0.5 to 0 s**	**0 to 1 s**	**0 to 1.5 s**	
Sub1	80	91	80	45/10
Sub2	78	95	96	41/11
Sub3	97	81	97	40/16
Sub4	66	95	70	36/9
Sub5	84	97	84	49/9
Sub6	66	72	86	48/5
Average	78.5 (10.7)	88.5 (9.05)	85.5 (9.27)	43/10

*The mean scores given by high-frequency power spectral classifiers trained from the pre- and two during-stimulus intervals in each subject. Overall accuracy given in the last row are the accuracies over all subjects considered for classification.*

*REM, remembered; FOR, forgotten.*

**TABLE 3:** Results of the t-test for the difference between the remembered and forgotten conditions.

**Time**	**Band**	**Feature set**	**t-value**
Pre-stimulus interval	Low gamma (30–60 Hz)	ITG (L)	2.828**
		MTG (R)	1.618*
		MTG (L)	1.315*
		STG (R)	2.358*
	High gamma (60–150 Hz)	ITG (R)	2.158**
		PFC (R)	1.785*
		MTG (R)	2.215**
		IPL (R)	1.582*

**Time**	**Band**	**Feature set**	**t-value**

During-stimulus interval	Low gamma (30–60 Hz)	ITG (L)	3.515*
		MTG (R)	2.357**
		MTG (L)	1.298**
		STG (R)	1.685*
	High gamma (60–150 Hz)	ITG (R)	2.553**
		PFC (R)	1.699*
		MTG (R)	2.288*
		IPL (R)	1.453*

*p < 0.05, **p < 0.01.

*ITC, inferior temporal cortex; PFC, prefrontal cortex; IFG, inferior frontal gyrus; IPL, inferior parietal lobule; L, left; R, right.*

*The t-statistic values and regions of the selected features. The most informative frequency values with the top four ranked of t-statistics were selected as the features in each phase and frequency band. The pre-stimulus interval showed positive spectral SME in the low-gamma bands (i.e., 38–50, 35–48, 32–40, and 38–54 Hz, respectively) and in the high-gamma band (65–70, 81–90, 78–95, and 81–93 Hz, respectively). The during-stimulus interval showed positive spectral SME in the low-gamma (38–59, 35–54, 42–54, and 38–55 Hz) and high-gamma band (82–90, 78–150, 80–150, and 82–109 Hz, respect.*

### Comparison With Other Approaches

Four other approaches were implemented and tested on the outperformed data set of the during-stimulus interval (0–1 s) using the same experimental protocols for comparison. As shown in Supplementary Table 1, two different classifiers performed over chance level predictions. Among these approaches, SVM achieved higher accuracy but was similar to linear discriminant analysis (LDA) and Fisher linear discriminant analysis (FLDA), which are effective methods that classify features with linear separability.

## Discussion

This study demonstrated that neocortical HFA (i.e., gamma power) predicted successful memory encoding, with average prediction accuracies of 78.5 and 88.5% for the pre-stimulus and during-stimulus intervals, respectively. The prediction rate improved by 10% when using during-stimulus intervals from the pre-stimulus interval. The majority of above-chance predictions were associated with activity in lateral temporal cortical regions, suggesting that cortical HFA values predict memory encoding.

To date, there have been no studies comparing data from pre- and during-stimulus intervals to predict subsequent memory formation using cortical ECoG activity. In accordance with our findings, several scalp EEG studies have demonstrated that pre- or during-stimulus electrophysiological brain activity predicted memory formation or subsequent memory. For instance, both neural signals before (Otten et al., 2010) and during an event (Sun et al., 2016) enabled the distinction of remembered events from forgotten ones. Indeed, by combining information from pre- and during-stimulus periods with single-trial-based classification methods, high-resolution surface EEG recordings predicted subsequent memory (Noh et al., 2014).

This is the first study to demonstrate the efficacy of HFA in cortical regions for memory prediction. Our data revealed specific gamma activity from different sub-bands (low gamma, 30–60 Hz; high gamma, 60–150 Hz) depending on cortical region during the 200–300 ms after stimulus presentation or later, which typically indicates induced activity (Basar-Eroglu et al., 1996; Tallon-Baudry et al., 1998). Studies have demonstrated that HFA may play a role in encoding information. A previous study reported an increase in gamma power (20–80 Hz) in subjects performing a visual delayed-matching-to-sample task while memorizing information, particularly in the occipitotemporal and frontal regions (Tallon-Baudry et al., 1998). In fMRI studies, the positive gamma SME in lateral temporal regions mirrors the localization of the positive SME (Wagner et al., 1998; Davachi et al., 2001; Reber et al., 2002). Similar to our findings, iEEG recordings of subjects during the encoding of a verbal noun memory task revealed that gamma oscillations (44–64 Hz) in the left temporal and frontal cortices predicted successful encoding of new verbal memory (Sederberg et al., 2007).

The majority of significant HFA during pre- and during-stimulus periods was observed in the lateral temporal cortices. The functional relevance of lateral temporal cortical activity in memory formation is unclear. The lateral temporal cortical regions play a functional role in memory formation, as this is a critical region in episodic memory processing (Chao et al., 1999). In humans, neuronal activity in the lateral temporal cortex subserves the encoding of verbal material networks (Ojemann and Schoenfield-McNeill, 1998; Ojemann et al., 2002, 2009). Previous functional imaging studies support temporal changes in cortical activity during the encoding stage of explicit verbal memory (Casasanto et al., 2002; Fletcher and Tyler, 2002). In line with this, a recent direct human brain stimulation study demonstrated causality between the direct stimulation of the lateral temporal cortices and verbal memory encoding (Kucewicz et al., 2018). Our recent hippocampal stimulation study also revealed that successful memory encoding involves the temporal cortex, which may act in concert with the hippocampus (Jun et al., 2019). Collectively, these findings suggest that the lateral cortex supports the functional connectivity underpinning memory formation.

The present study demonstrated that the pre- and during-stimulus brain activity in the lateral cortex could be used to distinguish subsequently remembered trials from forgotten trials. This indicates that the characterized high-frequency neural correlates of the lateral temporal cortex can predict subsequent memory. In this regard, investigating neural high-frequency oscillatory changes in memory-related temporal neocortical regions that modulate memory processes may provide insight into our understanding of the neural basis of episodic memory.

## Data Availability Statement

The datasets generated for this study are available on request to the corresponding author.

## Ethics Statement

The studies involving human participants were reviewed and approved by Seoul National University Hospital. The patients/participants provided their written informed consent to participate in this study.

## Author Contributions

SJ, JK, and CC contributed to the study design and wrote the article. SJ and CC performed the study. SJ and JK analyzed the data. JK and CC obtained funding. All authors contributed to the article and approved the submitted version.

## Conflict of Interest

The authors declare that the research was conducted in the absence of any commercial or financial relationships that could be construed as a potential conflict of interest.
