# Supplementary material for: Prediction of Successful Memory Encoding Based on Lateral Temporal Cortical Gamma Power
Source: Front Neurosci. 2021 May 25;15:517316. doi: 10.3389/fnins.2021.517316 (PMC8185029; doi:10.3389/fnins.2021.517316)
Supplement: Supplementary file 1 [file Table_1.docx]

Supplementary Table S1. Prediction performance of linear discriminant analysis.

| Approach | Average Accuracy (%) | Max Accuracy (%) |
| --- | --- | --- |
| LDA | 78 | 82 |
| FLDA | 80 | 82 |

Another classification using LDA and FLDA were trained to learn the high-frequency features of pre- and during-stimulus SME. The overall prediction of max accuracy achieved 82 %.
